# Temperature Variability and Occurrence of Diarrhoea in Children under Five-Years-Old in Cape Town Metropolitan Sub-Districts

**DOI:** 10.3390/ijerph13090859

**Published:** 2016-08-29

**Authors:** Gentille Musengimana, Fidele K. Mukinda, Roderick Machekano, Hassan Mahomed

**Affiliations:** 1 Division of Community Health, Faculty of Medicine and Health Sciences, Stellenbosch University, Cape Town 7505, South Africa; drfidelekanyimbu@gmail.com; 2 Centre for Health Systems and Services Research and Development, Faculty of Medicine and Health Sciences, Stellenbosch University, Cape Town 7505, South Africa; 3 Center for Evidence Based Health Care, Biostatistics Unit, Faculty of Medicine and Health Sciences, Stellenbosch University, Cape Town 7505, South Africa; rhoderick@sun.ac.za; 4 Metro District Health Services, Western Cape Government: Health, Cape Town 7505, South Africa

**Keywords:** temperature, diarrhoea, climate, infectious diseases

## Abstract

This paper describes the relationship between temperature change and diarrhoea in under five-year-old children in the Cape Town Metropolitan Area (CTMA) of South Africa. The study used climatic and aggregated surveillance diarrhoea incidence data of two peak periods of seven months each over two consecutive years. A Poisson regression model and a lagged Poisson model with autocorrelation was performed to test the relationship between climatic parameters (minimum and maximum temperature) and incidence of diarrhoea. In total, 58,617 cases of diarrhoea occurred in the CTMA, which is equivalent to 8.60 cases per 100 population under five years old for the study period. The mixed effect overdispersed Poisson model showed that a cluster adjusted effect of an increase of 5 °C in minimum and maximum temperature results in a 40% (Incidence risk ratio IRR: 1.39, 95% CI 1.31–1.48) and 32% (IRR: 1.32, 95% CI: 1.22–1.41) increase in incident cases of diarrhoea, respectively, for the two periods studied. Autocorrelation of one-week lag (Autocorrelation AC 1) indicated that a 5 °C increase in minimum and maximum temperature led to 15% (IRR: 1.46, 95% CI: 1.09–1.20) and 6% (IRR: 1.06, 95% CI: 1.01–1.12) increase in diarrhoea cases, respectively. In conclusion, there was an association between an increase in minimum and maximum temperature, and the rate at which diarrhoea affected children under the age of five years old in the Cape Town Metropolitan Area. This finding may have implications for the effects of global warming and requires further investigation.

## 1. Introduction

Diarrhoea is among the main causes of morbidity and mortality in children within the developing world [1]. Mortality associated with diarrhoeal disease among children under the age of five years old residing in developing countries was 2.50 million deaths per year worldwide based on a review published in 2003 of studies conducted between 1992 and 2000 [1]. In 2010, approximately 1.70 billion episodes of diarrhoea occurred, of which 36 million cases progressed to severe episodes; in 2011, there was an estimated 700,000 deaths attributed to diarrhoea in children under five years of age within the developing world [2]. Although a reduction in mortality has been documented, 15% of child deaths were still due to diarrhoeal disease [3].

The UN Millennium Development Goal 4 (MDG4) of reduction in mortality of children under the age of five years old by two-thirds between 1990 and 2015 has not been met in many regions and Sub-Saharan Africa (SSA) is among these [4]. SSA failed to meet the MGD4 because of an escalation in under-five mortality from four million in 1990 to 4.40 million in 2008 in this region (diarrhoeal disease making an important contribution to this as indicated above) [4].

According to the (2007) Intergovernmental Panel on Climate Change (IPCC) report, there is an expected increase in the global annual average temperature which could amount to 1.8–6.4 °C by 2100, which could result in increased temperatures and rainfall in many areas of the globe, causing significant temperature variability in the future [5]. It was reported that with a change in climatic parameters such as average temperatures and rainfall, the rate of certain health conditions i.e., thermal stress and infectious diseases increases [6]. It is worth noting that children under five years old are susceptible to the problem of climate-sensitive diseases with estimates ranging from 10% to 20% of populations in areas with limited capacity to manage the health impact of climate change [7]. Increasing evidence has emphasized the seasonal relationship between the peak of diarrhoea occurrence and climatic factors such as the rainy season and high temperatures in developing countries [8,9,10,11,12]. The link existing between climate parameters and diarrhoeal disease can be expected to fluctuate with different causal agents such as rotavirus, norovirus, *Giardia*, *Cryptosporidium* and pathogenic *Escherichia coli*, *Campylobacter* and *Salmonella* [13].

According to the World Health Organization (WHO), change in diarrhoeal disease incidence is among the health related problems expected as a result of climate change in the future [14]. Studies have shown that a higher population growth in Africa compared to other developing areas and the problem of climate change could result in high diarrhoeal disease mortality in children [15,16]. However, while climate change is predicted to cause an increasing global burden of diarrhoeal diseases, much is still to be investigated about climate factors, particularly in Africa, due to the inability to safely store health data and errors in data recording and archiving as a result of weak health infrastructure. It is important to understand climate variability as a risk factor for infectious diseases and to consider it as a foundation for climate change awareness. This is an area that is in urgent need of research in Africa [17].

Black et al. reported in 2010 that, in South Africa (SA), the number of children affected by diarrhoeal disease was around 72,553 cases per year and that about 6293 children die from the disease [3]. In addition, the 2012 report on under-five mortality statistics in SA using 1987–2007 data estimated that under-five mortality was about 71.8 per 1000 live births in SA, and that diarrhoea occupied 10% of the causes of death in children under five years of age [18]. Maclntyre and De Villiers in 2010 estimated that 24% of deaths in SA children within the age range of one to five years old was due to diarrhoeal diseases [19]. With regard to climate change and diarrhoeal diseases in SA, Thompson et al. found that diarrhoea occupied the first place among prevalent infectious diseases in the Limpopo province of SA. The overall burden of infectious diseases was 42.4%, and it was significantly associated with temperature variation in Limpopo Province of SA [20]. However, there is a need to further investigate the role of climate in the occurrence of diarrhoea and to further report on the mechanism of seasonality of diarrhoeal disease.

In the Western Cape, a SA province, the warm dry months between November and May offer conditions that favor the fast spread of pathogens causing diarrhoeal diseases. This season is known as the Diarrhoeal Disease Season (DDS) and it is marked by substantial increases in the number of patients with dehydration due to diarrhoea seen at health services across Cape Town, predominantly in children aged less than five years. The Western Cape Provincial Health Department plans annually to mitigate and reduce morbidity and mortality due to diarrhoea in the region. However, diarrhoeal disease still remains of major public health concern with about 25,000 to 30,000 cases expected each season in children aged less than five years. With regards to the population at risk among under five years old in the Cape Town Metropolitan Area (CTMA), diarrhoeal disease mainly affects children from poor families who live in informal settlement communities where there is a lack of basic services and poor access to health services.

Although recent studies have reported that temperature change could be among the risk factors promoting the spread of infectious diarrhoea [8,9,10,11,12], little evidence with regard to the reported association is available in South Africa. According to our knowledge, no study has been done assessing the relationship between environmental temperature variation and the childhood burden of diarrhoea in Cape Town. The climate in Cape Town differs from other parts of SA where rainfall occurs mainly in summer. This study aimed to explore the possible association between temperature variability and the high incidence of acute diarrhoea in Cape Town. Specific objectives were to verify the reported burden of paediatric diarrhoea in children under five years old from Cape Town metropolitan sub districts, to evaluate statistically the relationship between diarrhoea incidence and temperature and to determine if this relationship varied at subdistrict level. By gaining more in depth knowledge of the factors influencing the diarrhoeal disease peak in Cape Town, control efforts can be better informed and prepared, given what the potential effects of global warming might be.

## 2. Methodology

### 2.1. Study Design and Setting

A cross-sectional study was conducted using data from primary health care facilities and from the South African Weather Service to investigate the relationship between acute diarrhoea and change with temperature. The study setting was the Cape Town Metropolitan Area (CTMA) in the Western Cape Province of South Africa. This consisted of eight different sub-districts namely: Northern, Southern, Western, Tygerberg, Klipfontein, Mitchells’ Plain, Eastern and Khayelitsha.

According to the 2013 mid-year population census, the City of Cape Town had a population of 3,740,026 [21]. The CTMA is located in the South Western Peninsula of the Western Cape Province which is partially surrounded by the Atlantic Ocean. The CTMA has a Mediterranean climate whereby the majority of rainfall occurs during the cold winter months with an average daytime temperature range of 8.5 °C (minimum) and 18 °C (maximum); and a hot dry summer with an average daytime temperature around 16 °C (minimum) and 26 °C (maximum) [22]. According to the National Census of 2011, 87% of households in Cape Town had access to potable water and 90% had access to flush toilets with 20% of the population living in informal dwellings.

### 2.2. Study Population and Data Collection

One set of data was obtained from the public health services surveillance databases. It consisted of all children under the age of five, who reported with acute diarrhoea to the public sector run primary health care (PHC) facilities in the CTMA (confirmed by the health professionals working at these facilities, over the study periods of November 2012 to May 2013, and November 2013 to May 2014). These data on diarrhoeal episodes were compiled in an anonymised aggregated format (captured by data clerks and compiled by surveillance officers) by the Western Cape Department of Health (WDoH) and City Health Department of the City of Cape Town municipality. These facilities keep track of all presenting cases of diarrhoea from November to May on a weekly basis and from June to October on a monthly basis every year. Only cases of diarrhoea for children less than five years of age were captured electronically by the health services. For this study, the following variables were considered: name of clinic, week of the year, and sub-district name. However, the dataset did not include hospital data and some cases may have been missed i.e., those who may have bypassed the PHC facilities and presented directly at hospitals. The provincial authorities who provided the data checked the dataset for missing data and outliers and corrected these prior to the data being made available to the researcher. 

The second set of data used in this study were temperature data (daily minimum and maximum) provided by the South African Weather Service. This office keeps records of climate information such as daily minimum and maximum temperatures as well as precipitation. Given that diarrhoeal data were aggregated weekly, daily temperature data were collated into weekly data by calculating the mean temperature using the same dates of the weeks for which the diarrhoea dataset was collected. With regard to temperature data, high resolution data (individual data for each sub-district) were not available. Weather data for four stations i.e., Cape Point, Robben Island, Atlantis and Cape Town, were available for use. However, there was not much evidence of variation in temperature among the stations and, for this reason, data from the Cape Town weather station were used given that this station was closest to the study setting.

### 2.3. Statistical Analysis

The data were aggregated by week and period of data collection, as well as by sub-district level. Descriptive statistical analyses were conducted and mean, standard deviation and total numbers were established per period and for the overall 14 months of the study period. Using the number of cases and estimated population at risk (Census 2011 projections as provided by Western Cape Government: Health) in the two different study periods, incidence risk ratios were calculated and used to display a graphical overview of the burden of diarrhoea. Incidence risk ratio is a relative measure used to compare the rates of events occurring at any given point in time. 

In addition, a graphical presentation displaying trends in temperature and diarrhoea incidence cases over time was done. In order to test any possible association between temperature and the occurrence of diarrhoeal disease cases, a mixed effects over-dispersed Poisson regression model was constructed and an incidence risk ratio (IRR) with corresponding 95% confidence interval (CI) was reported. This model was used because the dataset was based on count of number of cases per week and the variance was greater than the mean. The model also accounts for clustering of diarrhoea cases within sub-districts. The model is a generalized linear model that has the assumption that the outcome variable Y (diarrhoeal incidence) follows a Poisson distribution. The model also assumes that the logarithm of its anticipated value can be modelled using a linear combination of unknown parameters.

An additional analysis was done where the effect of temperature was lagged by one week based on the assumption that a week’s average temperature might be related to the number of cases in the following week. In addition, we accounted for potential autocorrelation of outcomes in a model with a week-lagged outcome as an explanatory variable. All the analysis were done by adjusting for sub-district as a cluster and for each sub-district separately to evaluate if the association might be different in some sub-districts compared to others. The analysis was done using Stata version 14 (StataCorp, LP, College Station, TX, USA) and R software version 3.2.1 (R Foundation for statistical computing, Vienna, Australia). 

The study protocol was approved by the Health Research Ethics Committee of Stellenbosch University (Ref: S14/10/202), the City of Cape Town Health Research Committee (Ref: 10468), and the Western Cape Government: Health (Ref: WC_2015RP17_841). 

## 3. Results

### 3.1. Descriptive Statistics of Diarrhoea Disease and Climatic Variables

**Figure 1:**
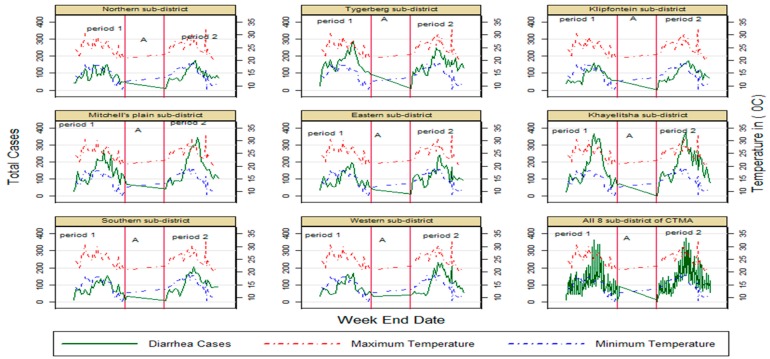
Trend in diarrhoea cases with change in min and max temperature in Cape Town Metropolitan Area CTMA sub-districts and in all eight sub-disricts. Period 1: November2012–May 2013; period 2: November 2013–May 2014, (**A**): period between period 1 and period 2 when no data were present for analysis (June 2013–October 2013).

Table 1 reflects the number of diarrhoeal cases, incidence rates and temperature ranges combined and over the two separate periods of seven months (November 2012–May 2013 represented by period one and November 2013–May 2014 represented by period 2) in the CTMA. Overall, 58,617 cases of children under the age of five were seen during the study periods. The incidence rates and temperatures means and ranges were similar for the two periods that include the summer months. Data for the winter months are not included, but diarrhoea incidence is much lower in those months and the downward trend towards the winter months is visible in Figure 1. 

### 3.2. Trend of Temperature and Incidence of Diarrhoea

**Figure 2:**
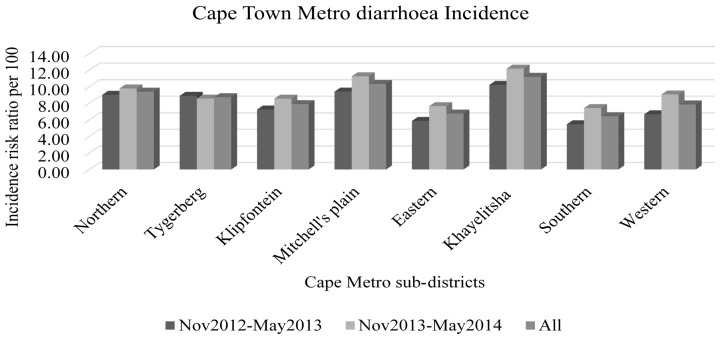
Distribution of diarrhoea incidence (number of cases/population at risk times 100) in different Cape-Metro sub-districts over the two selected periods and combined.

Trends of climatic parameters and incidence of reported diarrhoeal disease was done per sub-district and overall over the 14-month study period (Table 2, Figure 2). The relationship between temperature variation and diarrhoea incidence is displayed in Figure 1, which shows an overall increase in cases of diarrhoea as maximum and minimum temperatures increase during the study periods and a corresponding decrease as temperatures fall.

### 3.3. Poisson Regression Model

Table 3 shows the results of a Poisson model considering a cluster adjusted effect of a 5 °C increase in minimum and maximum temperature and demonstrates a significant effect of a 5 °C increase in both minimum and maximum temperature on incident cases of diarrhoea in children under 5 years, IRR 1.39 (95% CI: 1.30–1.48) (minimum temperature) and IRR 1.31 (95% CI: 1.22–1.40) (maximum temperature). The IRRs represent a 39% (95% CI: 30%–48%) and a 31% (95% CI: 22%–40%) increase in cases for minimum and maximum temperatures, respectively, for each five-degree increase. The cluster specific effect is different in some sub districts compared to others, but these differences were not statistically significant (Table 3). 

The cluster adjusted effect of 5 °C increase in minimum and maximum temperature with autocorrelation using a lag of one week (AC1), shows IRR 1.15, 95% CI: (1.09–1.20); IRR 1.06, 95% CI: (1.01–1.12), respectively (Table 3), but this effect is less than is the case with a simultaneous comparison. The IRRs represent a 15% (95% CI: 9%–20%) and 6% (95% CI: 1%–12%) increase in incident cases, respectively. It was also evident that the increased risk of diarrhoea, which is attributable to increased minimum and maximum temperatures with a one-week lag, is different across sub-districts, but the difference was not statistically different amongst the subdistricts (Table 4). Eastern and Western subdistricts did not achieve a significant association (confidence interval included one) for diarrhoeal incidence while all the others did for minimum temperature. Only Klipfontein subdistrict achieved a significant association for maximum temperature. The association was thus not as strong when a one-week lag was evaluated.

## 4. Discussion

This study evaluated the effect of environmental temperature variability on diarrhoeal occurrence in children under the age of five years old in the CTMA within two selected consecutive summer periods. A high number of diarrhoeal cases was observed during the summer/autumn months of November to May of the periods 2012/2013 and 2013/2014. The number of diarrhoeal cases is associated with environmental temperature variation and while temperature had varying strengths of associations in different sub-districts. However, the differences in the relationship between temperature and diarrhoea incidence in different sub-districts were not statistically significant. A one-week lag analysis showed a lower incidence risk ratio, but there was still a significant association with temperature overall. These findings are in line with those reported by the World Health Organization (WHO), emphasizing that global climate change may adversely affect the incidence of diarrhoeal disease as a consequence [14].

In Bangladesh, a seasonal increase in cases of diarrhoea was observed in the years 2000–2006 [11]. In this study, the results showed that an increase in one hot day led to an increase in daily diarrhoeal cases by 0.80% to 3.80% [11]. We observed that a 5 °C increase in minimum and maximum weekly temperatures could lead to an increase in cases of diarrhoea by 40% and 31%, respectively, in each season (period 1 and 2). This association was not unexpected as it has been computed in other studies [23]. In other studies, it was found that increased environmental temperatures were associated with an increase in visits to emergency departments, clinic visits and number of counted cases of diarrhoeal diseases in children [24,25,26]. In addition to these, the relationship between diarrhoea incident cases and temperature has been documented in other areas such as Peru, Fiji and Dhaka where a 1 °C increase in ambient temperature could result in 8%, 3% and 6% increases in diarrhoeal cases, respectively [9,10,27]. This is equivalent to what we found in our study.

The impact of environmental temperature increase on the incidence of diarrhoea cases observed in the CTMA study may be a result of many factors. It could be due to the fact that high temperatures lead to increased exposure to bacterial and other parasites causing diarrhoea, meaning that the bacteria-related gastrointestinal diseases are more likely to occur in high temperatures because bacteria that contaminate food are more prevalent in the environment during summer [28]. This might relate to the theory that a wide number of diarrhoea causing pathogens propagate faster and survive more easily in higher temperatures, thereby increasing the probability of being exposed to contaminated food and water [29]. *Salmonella*, *Cholera* and *Escherichia coli* are reported to multiply more easily in higher temperatures [6,30].

Our study showed that an increase in environmental temperatures has a different effect in different sub-districts of the study area and that, in some sub-districts, the association is stronger than in others, suggesting that the effect of temperature may vary in different regions [31]. The differences were not statistically significant and our study might have been underpowered to detect such differences. Possible reasons for sub-district differences might be due to poverty in some areas and deficiencies in infrastructure and as a consequence, families having decreased access to uncontaminated water and appropriate sewage disposal [32]. In addition, poor hygiene in food handling, diminished accessibility to medical care and low education levels might be greater in some sub-districts than in others, thereby causing some under five-year-old children to be more affected by diarrhoeal diseases in certain areas [32]. The contribution of different causative pathogens to diarrhoeal disease varies depending on the area of residence and the risk of faecal oral transmission. Food and water contamination is high for children living in areas with poor sanitation [32]. Khayelitsha is an area known to have a lower socio-economic status compared to other areas of the CTMA. Census data has indicated that the percentage of unemployed people in Khayelitsha of 35.7% is high compared to the provincial unemployment level of 17%. A large number of people in this metro sub-district do not have access to basic services. Only 23% of households in Khayelitsha have access to household tap water compared to a 68% average for the Western Cape Province [33]. Other studies investigating the relationship between temperature and diarrhoea controlled for a range of factors [9,10,11,12,13,15,17,20,23,24,25,26,27,28,29]. Many focused on the impact of age, gender, seasonality and rainfall. A few examined locally relevant factors such as density and snowmelt. Others have included factors that few have mentioned but may be relevant to all studies such as socioeconomic factors, water availability, hygiene and sanitation status. Extreme weather such as heavy rainfall and heatwaves were examined in certain studies. These all suggest that care needs to be taken in how such studies are designed so that confounding factors are appropriately addressed.

The lagged effect of climatic factors on diarrhoea morbidity indicates that, in some instances, there is a lag period between the time of exposure and occurrence of diarrhoea. This is evident in the autocorrelation analysis where a 5 °C increase in minimum and maximum temperature resulted in an increase of 15% and 6% cases of diarrhoea the following week, respectively (Table 3). The lag effect was weak compare to the effect observed with simultaneous analysis; this could possibly be because the incubation period of the causative organism was shorter than a week. Though there is a weak lag effect, the lag effect may be a possible indication of the range of the incubation period between the week of exposure to high temperatures and occurrence of diarrhoea. Diarrhoea caused by entero-hemorragic strains have an incubation period of around two to eight days with a median of 3–4 days and a period of communicability of about three weeks in one-third of children [34]. Diarrhoeal disease as a result of rotavirus infections has a short incubation period of one to three days, but the virus can also be shed in faeces with a mean period of four days, which could explain the lagged period between the time of exposure and occurrence of diarrhoeal disease, although in immunocompromised children, it could be as long as >30 days [35,36]. It could be that some cases manifested later in the following weeks depending on the causal agent and its incubation period, but this was not assessed. However, the stronger association with simultaneous comparison compared to one-week lag suggests that the diarrhoea occurring in the CTMA mainly has a short incubation period of less than one week.

### Study Strengths and Limitations

One of the strengths of this study was the ability to use an existing dataset (surveillance data) collected by health facilities and weather services to evaluate the relationship between diarrhoeal disease in children under five years old and selected climatic factors (temperature). However, weekly aggregated data could have diluted the effect measure. Although we used a mixed effects over-dispersed Poisson regression model to evaluate the possible relationship between paediatric diarrhoeal disease occurrences and temperature variability, some limitations exist such as age-specific characteristics, confounding factors like behavior patterns, vaccination coverage and the fact that children have different immune systems and dietary practices. In addition, a 40% and 32% increase in diarrhoea incident cases for every 5 °C increase in minimum and maximum temperature is high; this might be because only one explanatory factor (sub-district clustering) was adjusted for in the regression model. If other factors such as socio-economic, seasons, holidays, and demographic characteristics, accessibility to the health care facilities, parents’ education and age-specific characteristics of children in different sub-districts were adjusted for, the effect might have been different. Climatic factors alone may not be enough to explain the increased occurrence of diarrhoea in this population, given that the diarrhoea epidemic involves complex and critical interactions between intrinsic dynamic and extrinsic environmental factors. Furthermore, we could not analyze individual patient data. More detailed information on dietary behavior, vaccination coverage, age-specific characteristics, other environmental factors and diarrhoea causal agents are required to further explore the association between climatic factors and diarrhoea. We recommend that further studies be done using individual patient data and these other factors should be detailed there. Only two seasons of data have been analyzed, as long-term weekly data for diarrhoea were not available. Thus, caution needs to be used in applying the findings to the impact of long-term climatic change.

## 5. Conclusions

There is a seasonal increase in occurrence of diarrhoeal cases in children under the age of five years old in the CTMA. This appears to be related to variability in both minimum and maximum environmental temperatures. However, this association does not imply causation as many factors have to be considered in order to prove that the association is causal. To better study diarrhoea and factors associated with its occurrences, detailed environmental and climatic observations (such as rainfall, humidity, wind, access to water, access to sanitation) and detailed surveillance data (antigenic factors, whole-genome analysis) are required to improve the accuracy of predicting diarrhoea morbidity. All these are needed to be included in the model to explain the peak and trends in diarrhoea morbidity as observed in the CTMA. However, the results generated in this study could assist in coming up with a Department of Health Policy with respect to an early warning system when temperature changes are expected.

## Figures and Tables

**Table 1:** Temperature variations and incidence of diarrhoea in two study periods.

Climatic Variables	Period 1 *	Period 2 *	* Period 1 + 2
**Min Temperature**			
Mean (SD)	14.50 (3.20)	15.20 (2.70)	14.80 (2.90)
Range (Min, Max)	8.30 to 18.70	10 to 19.30	8.30 to 19.30
**Max Temperature**			
Mean (SD)	25.00 (2.60)	25.90 (2.90)	25.10 (2.70)
Range (Min, Max)	20.90 to 30.60	20.10 to 32.50	20.10 to 32.50
**Diarrhoea Incident Cases**			
N	27,031	31,586	58,617
Incidence/100, 95%	7.85/100, (7.76–7.96)	9.29/100, (9.19–9.39)	8.57/100, (8.50–8.63)
CI	Population at risk 344,238	Population at risk: 339,999	Population at risk: 684,237

* 7 months each period. 14 months for both; N: sample size; Min: Minimum; Max: Maximum; SD: Standard Deviation; CI: Confidence Interval (Period 1: November 2012–May 2013, period 2: November 2013–May 2014).

**Table 2:** Number of cases over a period of seven months (November 2012–May 2013 and November 2013–May 2014) with approximate population at risk in that period by sub-district. These figures were used to calculate incidence of diarrhoea presented in Figure 2.

Sub-Districts Names	November 2012–May 2013		November 2013–May 2014	
	Number of Cases	Population at Risk *	Number of Cases	Population Risk *
Northern	2642	29,288	2832	28,928
Tygerberg	4837	54,367	4599	53,698
Klipfontein	2580	35,617	3009	35,178
Mitchell’s Plain	4225	44,904	4992	44,351
Eastern	2924	49,805	3765	49,191
Khayelitsha	4814	47,099	5667	46,520
Southern	2433	44,536	3264	43,987
Western	2576	38,622	3458	38,147

* The population at risk was less in the later period than the earlier period based on estimates received from Statistics South Africa via the Western Cape Government: Health Department.

**Table 3:** Effect of 5 °C increase in minimum and maximum temperature.

Cluster/Sub-District	Minimum Temperature (5 °C Increase)		Maximum Temperature (5 °C Increase)	
	IRR	95% CI	IRR	95% CI
All (in total)	1.39	1.30–1.48	1.311	1.224–1.405
Northern subdistrict	1.38	1.19–1.60	1.29	1.11–1.51
Tygerberg subdistrict	1.31	1.16–1.47	1.24	1.09–1.41
Klipfontein subdistrict	1.42	1.21–1.67	1.35	1.14–1.59
Mitchell’s plain	1.42	1.18–1.70	1.29	1.07–1.57
Eastern subdistrict	1.39	1.16–1.66	1.29	1.07–1.55
Khayelitsha subdistrict	1.63	1.34–1.99	1.46	1.19–1.79
Southern subdistrict	1.48	1.24–1.78	1.39	1.16–1.67
Western Sub-district	1.46	1.16–1.83	1.33	1.05–1.67

All (in total): cluster adjusted effect for all sub-district in general; IRR: Incidence Rate Ratio; 95% CI: Confidence interval.

**Table 4:** Effect of 5 °C increase in minimum and maximum temperature with a one-week lag.

(AC 1) Cluster/Sub-District	Minimum Temperature (AC (1) of 5 °C Increase)		Maximum Temperature (AC (1) of 5 °C Increase)	
	IRR	95% CI	IRR	95% CI
All (in total)	1.15	1.09–1.20	1.06	1.01–1.12
Northern sub-district	1.17	1.01–1.35	1.07	0.92–1.24
Tygerberg sub-ditrict	1.15	1.04–1.26	1.08	0.97–1.19
Klipfontein sub-district	1.16	1.04–1.29	1.14	1.02–1.27
Mitchell’s Plain	1.15	1.02–1.31	1.08	0.95–1.22
Eastern sub-district	1.15	0.98–1.35	1.05	0.89–1.23
Khayelithsa	1.25	1.09–1.44	1.12	0.98–1.29
Southern sub-district	1.17	1.02–1.34	1.09	0.95–1.25
Western sub-district	1.13	0.96–1.34	0.95	0.79–1.12

AC1: Autocorrelation with 1 week lag; IRR: Incidence risk ratio; 95% CI: confidence interval.

## References

[1] Kosek M., Bern C., Guerrant R.L. (2003). The global burden of diarrhoeal disease, as estimated from studies published between 1992 and 2000. Bull. World Health Organ..

[2] Walker C.L.F., Rudan I., Liu L., Nair H., Theodoratou E., Bhutta Z.A., O’Brien K.L., Campbell H., Black R.E. (2013). Global burden of childhood pneumonia and diarrhoea. Lancet.

[3] Black R.E., Cousens S., Johnson H.L., Lawn J.E., Rudan I., Bassani D.G., Jha P., Campbell H., Walker C.F., Cibulskis R. (2010). Global, regional, and national causes of child mortality in 2008: A systematic analysis. Lancet.

[4] You D., Wardlaw T., Salama P., Jones G. (2010). Levels and trends in under-5 mortality, 1990–2008. Lancet.

[5] Solomon S. (2007). Climate Change 2007—The Physical Science Basis: Working Group I Contribution to the Fourth Assessment Report of the IPCC.

[6] McMichael A., Woodruff R., Hale S. (2006). Climate change and human health: Present and future risks. Lancet.

[7] Ebi K.L., Paulson J.A. (2007). Climate change and children. Pediatr. Clin. N. Am..

[8] Cairncross S., Feachem R. (1993). Environmental Health Engineering in the Tropics: An Introductory Text.

[9] Checkley W., Epstein L.D., Gilman R.H., Figueroa D., Cama R.I., Patz J.A., Black R.E. (2000). Effects of EI Niño and ambient temperature on hospital admissions for diarrhoeal diseases in Peruvian children. Lancet.

[10] Hashizume M., Armstrong B., Hajat S., Wagatsuma Y., Faruque A.S., Hayashi T., Sack D. (2007). Association between climate variability and hospital visits for non-cholera diarrhoea in Bangladesh: Effects and vulnerable groups. Int. J. Epidemiol..

[11] Wu J., Yunus M., Streatfield P., Emch M. (2014). Association of climate variability and childhood diarrhoeal disease in rural Bangladesh, 2000–2006. Epidemiol. Infect..

[12] Hashizume M., Armstrong B., Wagatsuma Y., Faruque A., Hayashi T., Sack D.A. (2008). Rotavirus infections and climate variability in Dhaka, Bangladesh: A time-series analysis. Epidemiol. Infect..

[13] Harley D., Bi P., Hall G., Swaminathan A., Tong S., Williams C. (2011). Climate change and infectious diseases in Australia: Future prospects, adaptation options, and research priorities. Asia Pac. J. Public Health.

[14] World Health Organization Protecting Health from Climate Change: Connecting Science, Policy and People. http://www.who.int/globalchange/publications/reports/9789241598880/en/.

[15] Bandyopadhyay S., Kanji S., Wang L. (2012). The impact of rainfall and temperature variation on diarrheal prevalence in Sub-Saharan Africa. Appl. Geogr..

[16] United Nations (2006). Population Division of the Department of Economic and Social Affairs of the United Nations Secretariat, World Population Prospects: The 2006 Revision and World Urbanization Prospects: The 2005 Revision.

[17] Alexander K.A., Carzolio M., Goodin D., Vance E. (2013). Climate change is likely to worsen the public health threat of diarrheal disease in Botswana. Int. J. Environ. Res. Public Health.

[18] Under-5 Mortality Statistics in South Africa: Shedding Some Light on the Trend and Causes 1997–2007. http://www.mrc.ac.za/bod/MortalityStatisticsSA.pdf.

[19] MacIntyre U.E., de Villiers F.P. (2010). The economic burden of diarrheal disease in a tertiary level hospital, Gauteng, South Africa. J. Infect. Dis..

[20] Thompson A.A., Matamale L., Kharidza S.D. (2012). Impact of climate change on children’s health in Limpopo Province, South Africa. Int. J. Environ. Res. Public Health.

[21] Statistics South Africa Mid-Year Population Estimates 2014. http://www.statssa.gov.za/publications/P0302/P03022014.pdf.

[22] Bradley J., Bradley L., Vidar J., Fine V. (2011). Cape Town, Winelands & the Garden Route.

[23] Bennett A., Epstein L.D., Gilman R.H., Cama V., Bern C., Cabrera L., Lescano A.G., Patz J., Carcamo C., Sterling C.R. (2012). Effects of the 1997–1998 El Niño episode on community rates of diarrhea. Am. J. Public Health.

[24] Chou W., Wu J., Wang Y., Huang H., Sung F., Chuang C. (2010). Modeling the impact of climate variability on diarrhea-associated diseases in Taiwan (1996–2007). Sci. Total Environ..

[25] Xu Z., Liu Y., Ma Z., Toloo G.S., Hu W., Tong S. (2014). Assessment of the temperature effect on childhood diarrhea using satellite imagery. Sci. Rep..

[26] Zhou X., Zhou Y., Chen R., Ma W., Deng H., Kan H. (2013). High temperature as a risk factor for infectious diarrhea in Shanghai, China. J. Epidemiol..

[27] Singh R.B., Hales S., de Wet N., Raj R., Hearnden M., Weinstein P. (2001). The influence of climate variation and change on diarrheal disease in the Pacific Islands. Environ. Health Perspect..

[28] Xu Z., Etzel R.A., Su H., Huang C., Guo Y., Tong S. (2012). Impact of ambient temperature on children’s health: A systematic review. Environ. Res..

[29] Rose J.B., Epstein P.R., Lipp E.K., Sherman B.H., Bernard S.M., Patz J.A. (2001). Climate variability and change in the United States: Potential impacts on water- and foodborne diseases caused by microbiologic agents. Environ. Health Perspect..

[30] Checkley W., Epstein L.D., Gilman R.H., Cabrera L., Black R.E. (2003). Effects of acute diarrhea on linear growth in Peruvian children. Am. J. Epidemiol..

[31] Kolstad E.W., Johansson K.A. (2010). Uncertainties associated with quantifying climate change impacts on human health: A case study for diarrhea. Environ. Health Perspect..

[32] Ryan M.O., Prado V., Pickering L.K. (2005). A millennium update on pediatric diarrheal illness in the developing world. Semin. Pediatr. Infect. Dis..

[33] Khayelitsha Transformation Research Project. http://stbweb02.stb.sun.ac.za/urdr/downloads/Khayelitsha.pdf.

[34] Chin J. (2000). Control of Communicable Diseases Manual.

[35] Dennehy P.H. (2000). Transmission of rotavirus and other enteric pathogens in the home. Pediatr. Infect. Dis. J..

[36] Pickering L.K., Bartlett A.V., Reves R.R., Morrow A. (1988). Asymptomatic excretion of rotavirus before and after rotavirus diarrhea in children in day care centers. J. Pediatr..

